# Fabrication of 3D PDMS Microchannels of Adjustable Cross-Sections via Versatile Gel Templates

**DOI:** 10.3390/polym11010064

**Published:** 2019-01-04

**Authors:** Pui Fai Ng, Ka I Lee, Mo Yang, Bin Fei

**Affiliations:** 1 Institute of Textiles & Clothing, Hong Kong Polytechnic University, Hong Kong, China; joanne.yu.ng@connect.polyu.hk (P.F.N.); Ka.I.LEE@connect.polyu.hk (K.I.L.); 2 Department of Biomedical Engineering, Hong Kong Polytechnic University, Hong Kong, China; Mo.Yang@polyu.edu.hk

**Keywords:** 3D microchannel, circular cross-section, hydrogel, PDMS, template molding

## Abstract

Flexible gel fibers with high stretchability were synthesized from physically cross-linked agar and covalently cross-linked polyacrylamide networks. Such gel material can withstand the temperature required for thermal curing of polydimethylsiloxane (PDMS), when the water in the gel was partially replaced with ethylene glycol. This gel template supported thermal replica molding of PDMS to produce high quality microchannels. Microchannels with different cross sections and representative 3D structures, including bifurcating junction, helical and weave networks, were smoothly fabricated, based on the versatile manipulation of gel templates. This gel material was confirmed as a flexible and reliable template in fabricating 3D microfluidic channels for potential devices.

## 1. Introduction

Three-dimensional (3D) microfluidic systems have demonstrated diverse advantages in micro-total analysis systems and bio-systems [1,2,3], chaotic micro-mixing [4,5] and optical manipulation [6,7]. Among many available methods, lithography is the most common approach to fabricate 3D microfluidic structures [8,9], where two-dimensional (2D) patterns are first generated and then assembled by aligning, stacking and sealing [2,4,10]. Although it allows various channel networks and interconnections, this fabrication technique always involves tedious and time-consuming multi-layer assembling. Frequent misalignment and sealing failures at the interface also make the process very challenging. The second common method is to combine 2D systems with flexible tubes [11] in which mechanical connections are usually long and not feasible for precise and compact interconnection. Stereolithography has been reported to overcome problems associated with conventional lithography techniques, providing a wide variety of channel shapes [12]. However, this process remains restricted by the high cost of instruments, the limited availability of photoactive materials, the sensitivity of light path to scattering and absorption media, and the strict requirement for competent operation skills.

Another challenge associated with microfluidic channel fabrication is the wide range of cross-sectional shapes. The microchannels prepared by lithography are usually restricted to rectangular cross sections. This geometry significantly disturbs the internal flow behavior of liquids [13], confining inertial particles to the sidewall under the domination of the corner effect [14]. Unlike rectangular channels, circular cross-sectional geometry allows radial focusing due to its symmetrical velocity profile, which proves the feasibility in vascular replication and flow simulation for biomedical engineering.

Microchannels with circular cross-section have been fabricated by modifying the photoresist masters via rapid prototyping [15], melting [16] or reflow baking [17]. Although these methods make it possible, the circular channel fabrication remains poor in quality control. Another strategy is to reshape rectangular channels by additional silicone oligomers polymerization [13,18,19], which provides only round corners without good symmetry.

To date, a number of reports have utilized the template molding technique to achieve a circular channel. Nylon thread [20], metallic wire [21,22] or optical fiber [23] were embedded in a polydimethylsiloxane (PDMS) platform as a template for well-defined microchannel fabrication. After molding, these templates can be removed mechanically to form well-constructed channels. However, these template materials are usually stiff and less flexible, causing channel distortion during template removal. Recently, a 3D printing technique to fabricate 3D microchannels has been reported [1]. A sacrificial agarose gel template is used for manufacturing artificial vasculature in photo-polymerized hydrogel. The gel template leads to complex microchannel patterns with minimal channel distortion due to its inherent softness. However, this thermo-reversible gel has a low sol-gel transition temperature of 32 °C, and is restricted to low-temperature operations. It only supports a photo-polymerizing matrix and limits the thickness of 3D structures below 1.0 cm. Therefore, this gel template technique still needs improvement to meet wide applications.

Herein, we introduce a new template material for PDMS molding: agar/polyacrylamide/ethylene glycol (agar/PAAm/EG) gel fiber. This gel template can withstand the temperature required for thermal curing and shows no adhesion to the surrounding PDMS. It can also be easily removed via manual pulling without channel distortion. Some representative microfluidic structures, including bifurcating junctions, helical and weave microchannels, were easily achieved. The presented gel template allows fabricating large scale 3D microstructures in a single step without tedious assembling processes.

## 2. Materials and Methods

### 2.1. Materials and Reagents 

Agar was obtained from Fisher Scientific (Hong Kong, China); acrylamide (AAm), ammonium peroxydisulfate (APS), *N*,*N*-methylenebisacrylamide (MBA), ethylene glycol (EG) and tris(2,2′-bipyridyl)dichlororuthenium (II) hexahydrate (Ru(bpy)_3_) were purchased from Sigma-Aldrich Co. (Hong Kong, China). Poly(dimethylsiloxane) elastomer kit (PDMS precursor, Sylgard^®^ 184) was obtained from Dow Corning Co. (Shanghai, China). All chemicals were used as received.

### 2.2. Gel Fiber Template Preparation

Agar/PAAm/EG gel fiber was synthesised by one-step free-radical polymerization of AAm in an agar solution of water and EG (H_2_O/EG = 100/0 − 30/70). Briefly, agar of 0.30 g, AAm of 0.034 mol and MBA (0.05 mol % of AAm) were dissolved in H_2_O/EG mixture at 85 °C under continuous stirring. A predefined amount of APS (0.2 mol % of AAm) was added into the above solution. The obtained mixture was transferred into a PTFE tube of round channel (Bohlender™, I.D. × O.D. = 1.0 × 2.0 mm, or 0.5 × 1.0 mm) (tubes of special cross-section shapes were self-made by folding book-covering polyester film of 0.15 mm thickness). The tubes were sealed tightly with clay to avoid water evaporation. AAm polymerization was carried out at 65 °C for 6 h. The resultant gel fiber was removed from the tube and used as a microchannel template.

To fabricate the junction gel template, photo-polymerizable AAm precursor was used as a glue for template connection. Briefly, AAm of 7.034 mmol, predefined amounts of MBA (0.9 mol % of AAm), APS (0.7 mol % of AAm), and Ru(bpy)_3_ (0.5 mol % of AAm) were dissolved in 4.5 mL water at room temperature under sonication. The obtained mixture was maintained in a dark environment to avoid reaction. The photo-polymerization of the as-prepared precursor was achieved under the Maxlite F25T8/850 XL daylight light tube (Distance: 10 cm, time: 5 min).

### 2.3. Microchannel Fabrication Using Gel Fiber Templates

**Figure 1:**
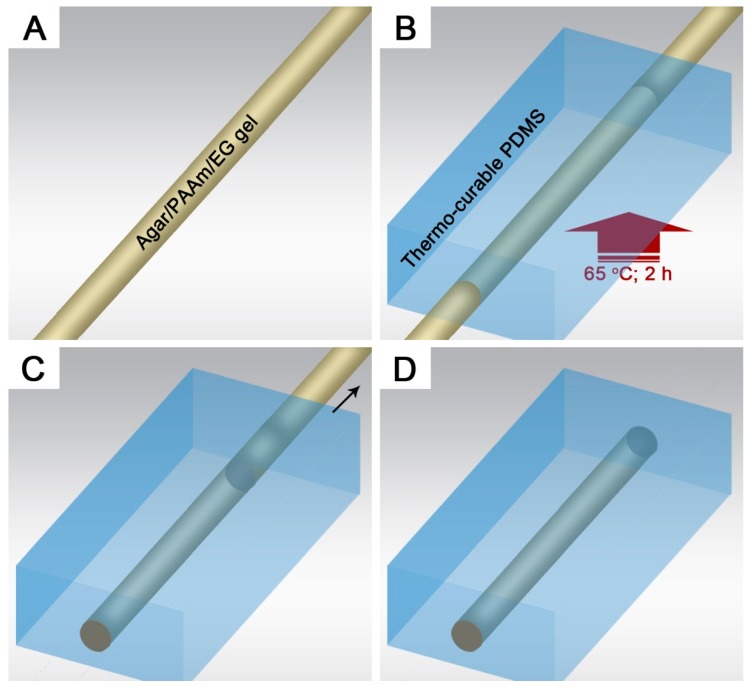
Schematic illustration of straight microchannel fabrication via template molding: (**A**) The gel fiber template with desired orientation; (**B**) Thermal curing of polydimethylsiloxane (PDMS) precursor casted over the gel template; (**C**) Pulling out the gel template from the PDMS matrix; (**D**) Resultant circular microchannel.

The microchannel was fabricated by the template molding technique, as shown in Figure 1. The gel fibers were positioned in desired orientations to present different channel patterns. After template shaping in a petri-dish, PDMS precursor with a base-to-curing agent ratio of 10:1 was casted into the petri-dish, degassed and cured at 65 °C for 2 h. The molded PDMS was demounted from the petri-dish and immersed in ethanol for 20 min, in which template shrinkage and channel swelling were performed. The embedded gel template was removed by manual pulling from the PDMS matrix, leaving behind a smooth microchannel. The PDMS was then air-dried and thus restored to its original state.

### 2.4. Characterization

Tensile strain at break (%), stress at break (MPa) and Young’ modulus (MPa) of gel fibers with different EG contents, from 0 to 50 wt %, were measured using a universal mechanical testing machine (Instron 5566, Shanghai, China) with a 10.0 N load cell. Samples (*n* = 6) were tested at a strain rate of 50.0 mm/min and a starting clamp distance of 10.0 mm [24].

The surface morphology of microchannels was observed using an optical microscope (Leica M165C, Hong Kong, China, with CCD camera Leica DFC290 HD). A scanning electron microscope (SEM, HITACHI TM 3000 Tabletop, Hong Kong, China, with ion sputter HITACHI E-1010) was also applied to check the smoothness of channel inner surface. For weave pattern observation, the channels were perfused with 0.01% (*w*/*v*) solutions of Levafix yellow and Levafix blue and were imaged with a digital camera (Canon A630, Hong Kong, China) to illustrate the channel separation.

## 3. Results and Discussion

### 3.1. Agar/PAAm/EG Gel Template Selection and Removal

This study aimed to introduce a new template material for microchannel fabrication within a PDMS matrix. Double network (DN) hydrogels based on covalently crosslinked PAAm have been reported by our group [24,25]. Such DN gels are tough and highly stretchable, and constitute a possible template for microfluidic channel fabrication.

**Figure 2:**
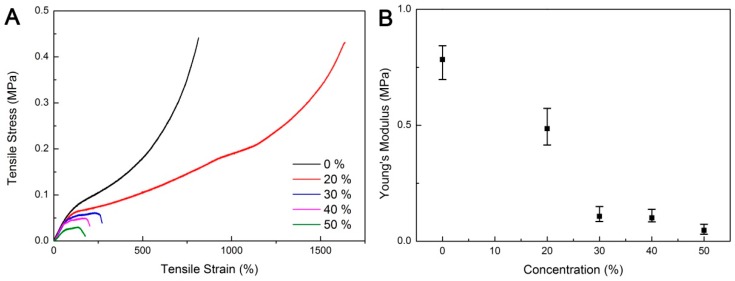
Mechanical properties of agar/PAAm/EG gels with different ethylene glycol (EG) contents: (**A**) typical tensile curves and (**B**) Average Young’s modulus.

**Figure 3:**
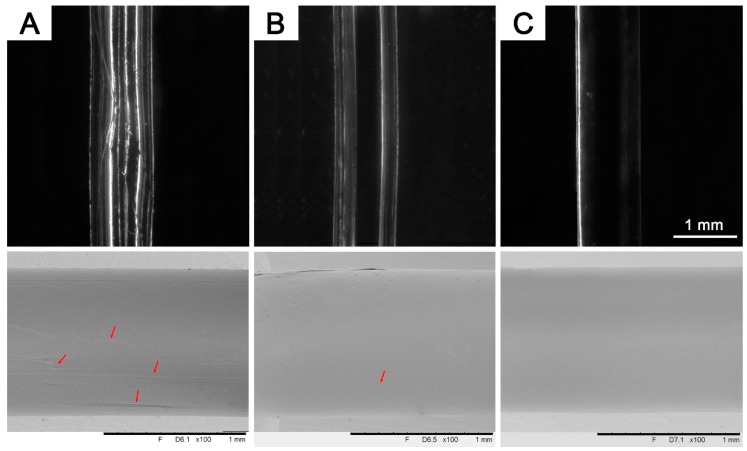
Microscopic (Top) and SEM (Bottom) images of microchannels fabricated using gel templates with different ethylene glycol (EG) contents: (**A**) 0 wt %, (**B**) 20 wt % and (**C**) 40 wt %, showing undesired defects on the inner surfaces (Red arrow).

Agar/PAAm DN hydrogel, composed of a physically cross-linked agar network and a covalently cross-linked polyacrylamide network [26], offers excellent flexibility and extensibility, as shown in Figure 2A. When the hydrogel template was incorporated in a PDMS matrix, it was ready to be manually pulled out smoothly. However, in practice, part of the PDMS precursor in contact with the hydrogel failed to polymerize under the normal curing process, resulting in groove-like defects at the interface (Figure 3A). It was ascribed to the substantial amount of vapor diffusion through the interface during thermal curing, since the water vapor can disturb the crosslinking of PDMS precursors. Upon thermal curing, the hydrogel water starts to evaporate and to diffuse into the PDMS matrix. Some of the Si–H groups in the precursor were consumed with water vapor through undesired reactions, which restrict the proper curing of PDMS. Because the hydrosilylation reaction of PDMS precursor is sensitive to water vapor that consumes the Si–H bond, the water vapor from the gel should be avoided as much as possible [27,28].

Surface smoothness is a crucial factor in microfluidics. In order to eliminate the side effect of hydrogel on PDMS curing, EG was introduced into the agar/PAAm templates to replace H_2_O. The gel has been successfully synthesized at a low H_2_O/EG ratio of 50/50. When the H_2_O/EG ratio became lower than 50/50, the EG evidently hindered the polymerization of AAm and caused the precipitation of agar, and thus resulted in the failure of DN hydrogel synthesis. Figure 3 shows the microscopy and SEM images of microchannels fabricated with the EG varying from 0 to 40%. With the EG introduction, a smoother inner wall surface was observed with less defects. Compared with water, EG has a considerably higher boiling point of 197 °C. The addition of EG raises the boiling points significantly and reduces evaporation during the thermal curing process. Elevated amount of EG in the template reduced water evaporation and suppressed the effect of water vapor on PDMS curing.

Hydrogel flexibility and stretchability are essential for the template removal. Figure 2A shows the tensile stress-strain curves of gels with different EG contents. The gels exhibited similar yielding strain after EG introduction. The fracture toughness of gels appeared to be affected by the presence of EG. The highest fracture toughness was obtained at an EG content of 20 wt %, with its tensile stress and strain at break values of 0.43 MPa and 1640%, respectively. Meanwhile, as indicated in Figure 2B, increase in EG content had lowered the Young’s modulus of the gel. When the EG content exceeded 20 wt %, the gel shows a significantly lower fracture toughness: Upon stretching, the samples exhibited obvious cracks and ruptured quickly at low strains. The introduction of EG may interrupt the AAm polymerization and thus deteriorate the gel mechanical properties. Acceptable modulus was observed for the gel with 20 wt % EG.

Since a flexible gel with high stretchability is preferred for template shaping and removal, only the agar/PAAm/EG hydrogel with 20 wt % EG was further used for the subsequent experiments. This tough gel material facilitated the convenient fabrication of microchannels at the thermal curing condition, and allowed easy removal of the template from the construct without damaging the channel surface.

### 3.2. Microchannel Morphology

**Figure 4:**
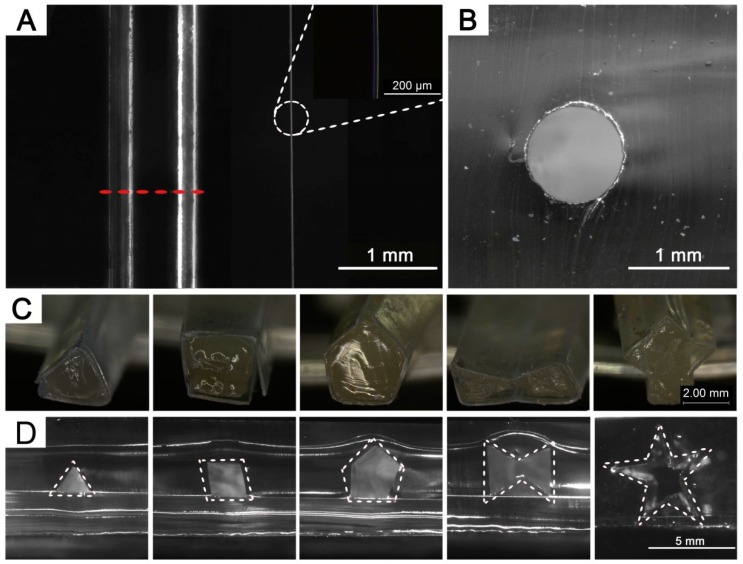
Microscopic images of the straight channels. (**A**) Microchannels with diameters of 1000 μm (Left) and 15 μm (Right). (**B**) Cross section of a microchannel showing the circular contour (Red dashed line). (**C**) Oblique view of polyester tube templates with hydrogel inside, showing shapes of triangle, rhombus, pentagon, hexagon and pentacle (left to right). (**D**) Cross sections of microchannels resulted from the above gel templates in polydimethylsiloxane (PDMS) matrix.

When the thermal curing of PDMS precursors was completed, the gel template was removed after immersing in ethanol, where the gel fiber shrinkage and the PDMS swelling (swelling ratio of 1.04) [29] facilitated easy template removal by reducing the frictional resistance inside the channel. The removal of gel template from the PDMS matrix generated an empty channel, which was smoother than those prepared using nylon threads [20] or metallic wires [22]. Channels with circular cross-section of various diameters (≥15 μm) were achieved by using the proposed gel templates, as shown in Figure 4A,B. Gel diameters can be reduced by gentle stretching to suitable length or immersing into ethanol for a certain time. Special attention should be paid to avoid undesirable over stretching that may result in cracks on the gel template and affect channel evenness. The gel template can also be used for channel shape control. Several other channels of different cross-section shapes have been designed and easily achieved as demonstrated in Figure 4C,D, including triangle, rhombus, pentagon, hexagon and pentacle shapes. In order to make the channel easier to observe, all the following channels will be made into 1000 µm diameter without special notation.

Furthermore, the proposed gel exhibited reversible swelling-shrinking properties in response to water content, which is advantageous to fabricate a microchannel with various diameters. Various geometrical changes, which are capable of continuous sampling, can be achieved by partially controlling its shrinkage rate.

**Figure 5:**
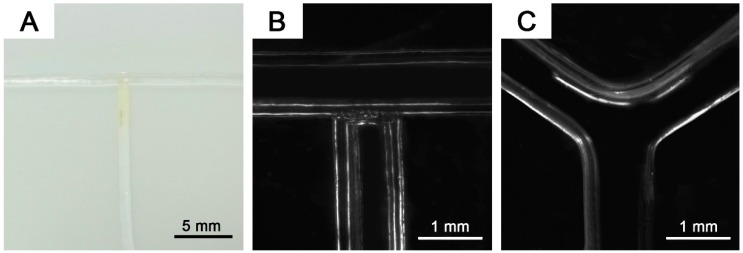
(**A**) Photo of the T-junction gel fiber template and microscopic images of (**B**) T- and (**C**) Y-junction microchannel.

Our gel template allowed the fabrication of various microchannel orientations, which were difficult to prepare using the conventional templates. The versatility of the template was demonstrated by fabricating a T- and Y-junction (Figure 5). Gel templates are connected via photo-polymerization of aqueous glue. The glue precursor was absorbed into the gel tip by tip-dipping. When the gel tip with glue came into contact with another gel fiber, the glue diffused through the interface. By exposing the interface to visible light of 532 nm for a few minutes, spontaneous joining was achieved by photo-polymerization without external force. With this approach, other types of interconnection between gel fibers can also be easily obtained. This joining is strong enough to survive the PDMS curing process. After template molding, the gel fibers were easily separated and removed by gently pulling them at the same time.

A bifurcating junction is a necessary structure for microfiber production by manipulating fluid-on-chip. The device with intersected circular channels assists the passive optimization of flow-focusing in 2D configuration, facilitating the control over the coaxial fluid flow [30]. With our gel templates, a multi-junction device can be easily fabricated without any bonding or aligning.

**Figure 6:**
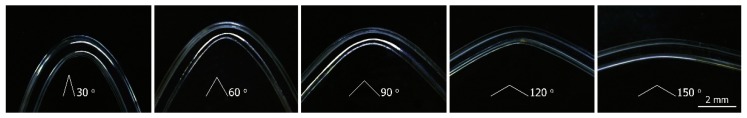
Microscopic images of channel corner with different bend angles.

A flexible template is particularly advantageous to complicated network fabrication. Various orientations can be accomplished by template bending. This process, however, causes deformation on common solid materials. Elongation occurs at the outer edge of the bend, causing a distortion of its cross section. At the inner edge of the bend, wrinkles are formed at a certain point due to compression. Therefore, a good template should resist deformation during bending. Our gel material possesses high elasticity and softness, which helps maintain its original dimension without surface wrinkles (Figure 6). Even when the gel was bended into a 30° angle, its cross-section and surface quality were well-maintained, thus supporting production of a smooth microchannel.

**Figure 7:**
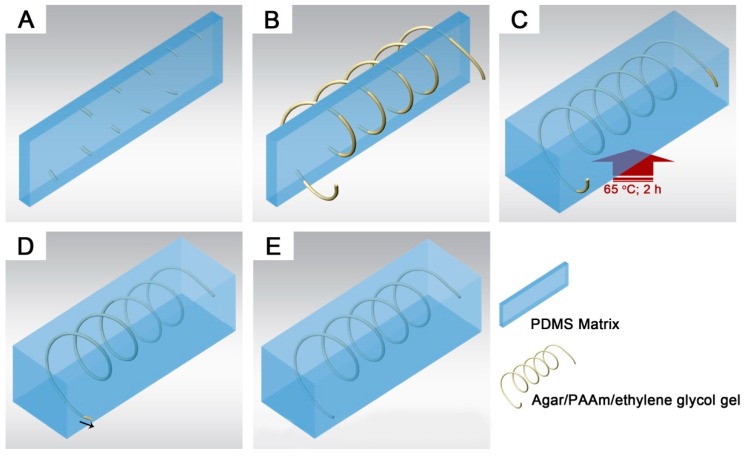
Schematic illustration of template molding. (**A**) Polydimethylsiloxane (PDMS) sheet with punctures; (**B**) Helical pattern of gel fiber fixed with punctured PDMS sheet; (**C**) Thermal curing of PDMS precursor casted over the gel template; (**D**) Pulling out of the gel template embedded in the PDMS matrix; (**E**) Resultant 3D helical pattern of circular microchannel.

**Figure 8:**
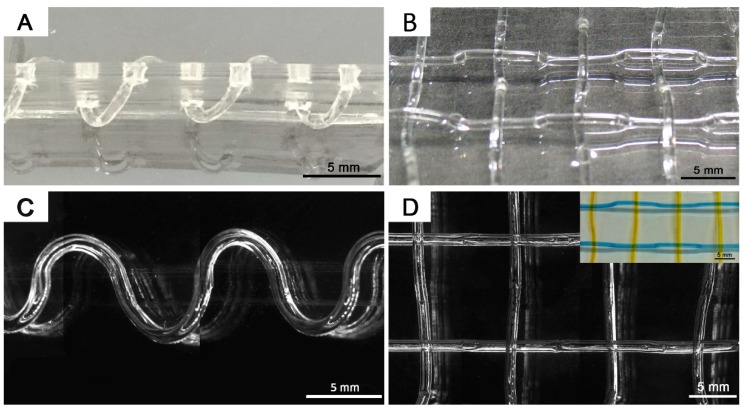
Photos of the gel fiber templates and microscopic images of the resultant microchannels: Helical (**A**) and weave (**B**) pattern of gel fiber template fixed on punctured polydimethylsiloxane (PDMS) sheet. Microscopic images of the resultant helical (**C**) and 3D weave network (**D**). Inset of two microchannels perfused with yellow and blue solutions, respectively.

Meanwhile, a good gel template should not be limited by any complicated 3D channel network. Our gel was flexible enough for simple fabrication of complex structures, as schematically depicted in Figure 7. A 3D pattern can be formed by fixing the fiber template on a punctured PDMS sheet. A single helical channel and a weave network were obtained by threading the gel templates through the hole-arrayed sheet, as shown in Figure 7. The resultant microchannels were completely separated without fluid leakage, that was often caused by layer misalignment or sealing failure in lithography methods. As shown in Figure 8D, colored solutions were introduced into the weave network through two inlets to visualize the channel separation. No color mixing was found in the weave pattern, indicating hermetical channels without leakage between each other.

This molding strategy facilitated the fabrication of complex 3D microchannels, which are essential to realize a 3D structure of chaotic fluidic mixer [31]. The 3D chaotic mixer was required to facilitate multiple layer alignment in the lithography approach. Multiple layer alignment was required to achieve 3D channel structure via lithography. However, our gel material can create this complex structure in a single molding step. Serpentine- [31] and helical-shaped [32] mixers were successfully demonstrated.

This gel material exhibited high flexibility, stretchability and healability in microchannel fabrication. It provides a promising template alternative and a straight-forward approach to create microchannels for versatile microfluidic devices.

## 4. Conclusions

We successfully introduced a highly stretchable gel material for microfluidic channel fabrication within the widely used PDMS matrix. The smooth curing of PDMS was guaranteed by partially substituting water with EG in the gel template, which resulted in high quality channels of various diameters and cross-sectional shapes. Based on flexible assembly and organization of these reliable templates, representative 3D microfluidic structures were manufactured in a single molding step, including junctions, helical, and weave patterns. This versatile gel material has potential applications in microdevice fabrication and scaffold engineering.
